# Intravascular papillary endothelial hyperplasia of the maxillary sinus extending into the contralateral nasal cavity

**DOI:** 10.1007/s00405-024-08499-y

**Published:** 2024-03-19

**Authors:** Masahiro Nakamura, Takashi Anzai, Erina Ishimizu, Karin Ashikawa, Ayako Inoshita, Yusuke Takata, Fumihiko Matsumoto

**Affiliations:** 1 Department of Otorhinolaryngology, Faculty of Medicine, Juntendo University, 2-1-1 Hongo, Bunkyo-Ku, Tokyo, 113-8421 Japan; 2 Department of Human Pathology, Faculty of Medicine, Juntendo University, Tokyo, Japan

**Keywords:** Intravascular papillary endothelial hyperplasia, Maxillary sinus, Transnasal endoscopic surgery, Epistaxis

## Abstract

**Introduction:**

Intravascular papillary endothelial hyperplasia (IPEH) predominantly occurs in the subcutaneous and dermal regions and rarely originates from the sinonasal mucosa.

**Case presentation:**

We report on the case of a 58-year-old male patient who presented with progressive bilateral nasal obstruction, left-sided epiphora, and intermittent epistaxis. Computed tomography revealed a soft tissue opacity in the left maxillary sinus with intersinusoidal nasal wall demineralization, extending into the surrounding ethmoid cells and the right nasal cavity through a contralateral deviation of the nasal septum. Contrast-enhanced T1-weighted magnetic resonance imaging further confirmed these findings. The IPEH originating from the maxillary sinus extended into the contralateral nasal cavity, and it was successfully removed using an endoscopic endonasal approach, avoiding overly aggressive treatment.

**Conclusion:**

This case report highlights the diagnostic challenges of IPEH in the sinonasal region and the importance of considering IPEH as a differential diagnosis in patients presenting with nasal obstruction, epiphora, and intermittent epistaxis.

## Introduction

Intravascular papillary endothelial hyperplasia (IPEH), or Masson's tumour, is an uncommon benign vascular lesion characterized by papillary formation within the vascular lumen. Initially described in 1923, it is considered a reactive process that often develops in a dilated vessel lumen, hematoma, or preexisting vascular lesion, with no reported malignant transformation [1]. While IPEH affects various parts of the body, cases involving the sinonasal region are rarely reported. We present an intriguing case of IPEH in the left maxillary sinus extending to the right nasal cavity through a contralateral deviation of the nasal septum, outlining the diagnostic and therapeutic challenges. This is the first report of IPEH arising from the maxillary sinus extending into the contralateral nasal cavity in which malignancy was ruled out during surgery, and the tumor could be removed minimally invasively using only transnasal endoscopic surgery.

## Case

**Fig. 1:**
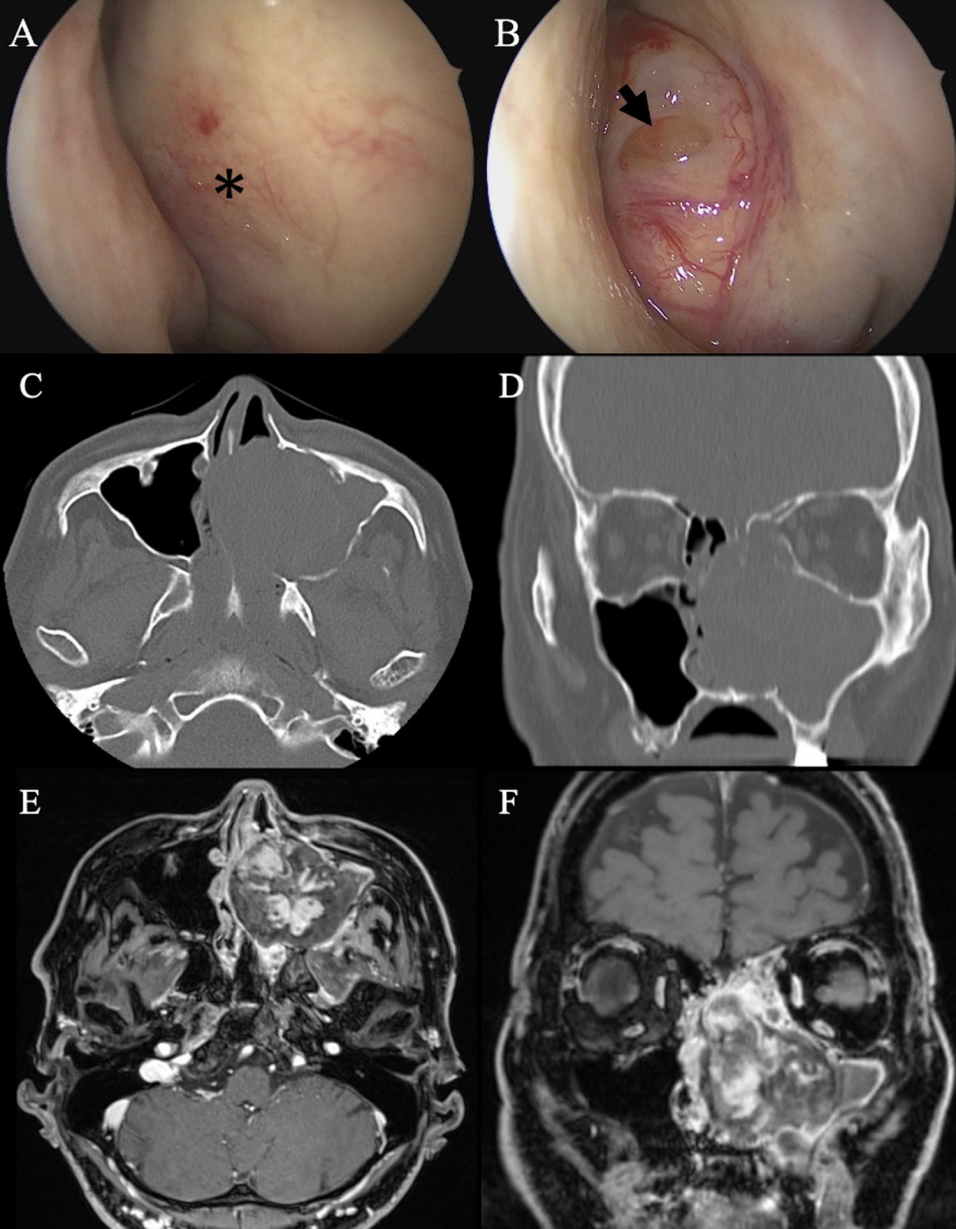
**A** Endoscopic view of right nasal cavity. The right nasal cavity was obstructed due to compression of the nasal septum: asterisk. **B** Endoscopic view of the lesion partially revealed a smooth, polypoid mass arising from the natural ostium of the left maxillary sinus: black arrow. Axial (**C**) and coronal (**D**) CT showing a soft-tissue, isodense mass in the peripheral area occupying the left nasal cavity. Multiple bony structures eroded by growth including the medial wall of the right maxillary sinus and a large portion of the nasal septum are visible. Axial (**E**) and coronal (**F**) MRI showing oedematous mucosa surrounding a heterogeneous tumor in the left nasal cavity

A 58-year-old male presented with progressive bilateral nasal obstruction, left-sided epiphora, and intermittent epistaxis. His medical history was unremarkable, with no history of facial trauma, bleeding tendency, allergies, or prior sinus surgery. He was a current smoker with a 35-pack-year history, smoking one pack of cigarettes daily for 35 years. The right nasal cavity was obstructed due to compression of the nasal septum; however, there was no evidence of tumour invasion into the mucosa (Fig. 1A). A polypoid nasal tumour was found in the left nasal cavity on transnasal endoscopy (Fig. 1B).

Computed tomography (CT) revealed a soft tissue opacity in the left maxillary sinus with intersinusoidal nasal wall demineralization due to tumour compression, extending into the surrounding ethmoid cells, and a significant septal deviation to the right (Fig. 1C, D).

Contrast-enhanced T1-weighted magnetic resonance imaging (MRI) confirmed these findings, exhibiting fluid and oedematous mucosa surrounding a heterogeneous tumour in the left nasal cavity (Fig. 1E, F).

The patient was admitted for endoscopic nasal tumour resection under general anaesthesia. The tumour was removed via transnasal endoscopic surgery without preoperative embolization of the sphenopalatine artery. Intraoperative frozen section analysis excluded features of malignancy, and the mass was removed entirely without surgical margins. The tumour peeled off the skull base, and there was no intradural invasion. There was no evidence of cerebrospinal fluid leakage. The total amount of intraoperative blood loss was 20 ml.

**Fig. 2:**
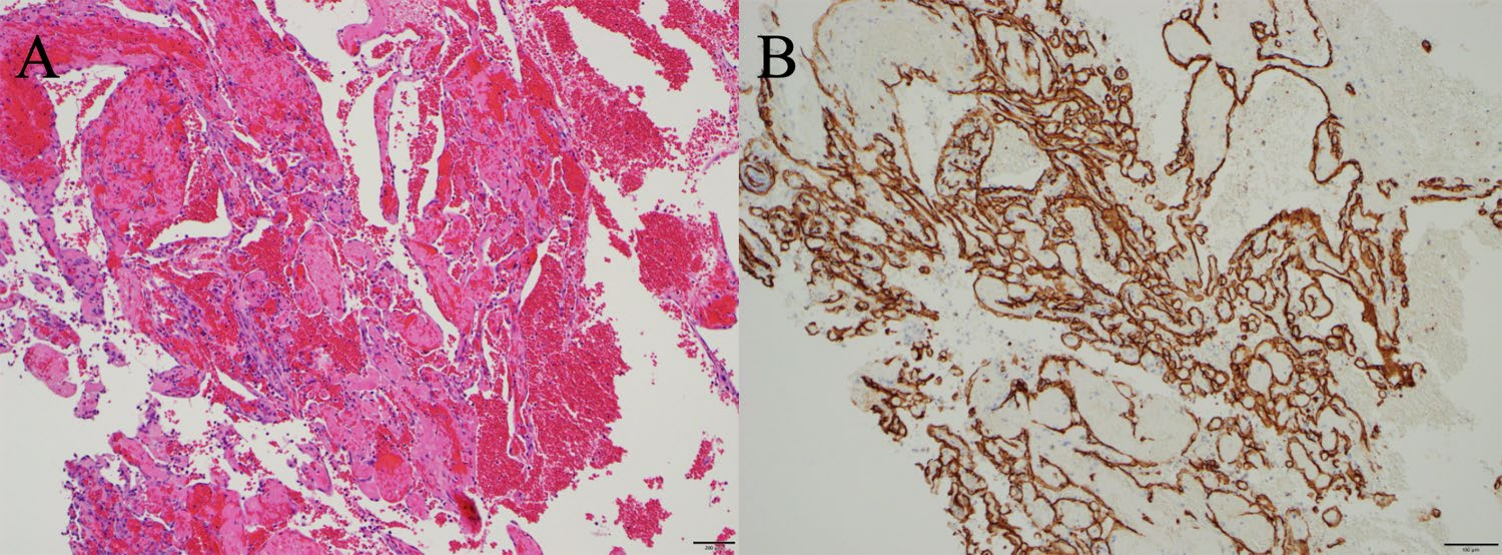
**A** Histopathologic image showing papillary hyperplasia of vascular endothelial cells (haematoxylin and eosin stain, × 100). **B** Immunohistochemical analysis showing endothelial positivity for CD34 (immunohistochemistry CD 34, × 100)

Histopathological examination revealed no malignancy; however, the papillary proliferation of the vascular endothelial cells was consistent with IPEH (Fig. 2A). CD34 positivity, consistent with the vascular endothelial cells, was also recognized (Fig. 2B).

The postoperative course was uneventful, with no symptoms such as empty-nose syndrome, nasal obstruction, epiphora, or recurrent bleeding.

## Discussion

Since Masson’s tumours were first described in 1923 [1], cases affecting almost every part of the human body have been reported. This tumour is often subcutaneous or dermal, with a predilection for the head, neck, fingers, and torso [2]. It is currently considered a reactive intravascular proliferation that develops in the lumen of a dilated vessel, hematoma, or preexisting vascular lesion [2, 3].

Additionally, approximately one-third of all cases originate in the head and neck, particularly in the neck, orbit, lip, pharynx, and mandible [3]. Tumours occurring in the paranasal sinuses or nasal cavity are rare, with only 14 reported cases to the best of our knowledge [4–15].

Symptoms vary and often include a combination of nasal congestion, epistaxis, postnasal drip, cheek pain, and frontal headaches.

These symptoms are attributed to lesions occurring in the nasal and sinus cavities that spread with bone demineralization, causing anatomical changes in the surrounding area. Of the 15 cases of sinonasal IPEH, including our report, nine were of the maxillary sinus [5, 7, 11, 13–15]. Among them, four cases complained of nasal obstruction [7, 11, 14, 15], and in addition to our case, only one case developed bilateral nasal obstruction [14]. Our patient complained of bilateral nasal obstruction because the tumour compressed the nasal septum and extended into the contralateral nasal cavity. Moreover, epiphora occurred due to obstruction of the left nasolacrimal duct. These symptoms disappeared entirely after tumour excision.

Radiologically, IPEH closely resembles angiosarcoma but has a much more favourable prognosis [16]. The differential diagnosis of IPEH is challenging primarily due to its resemblance to angiosarcoma; however, several key histologic features differentiate the two: (1) IPEH resides intravascularly, while angiosarcomas invade the surrounding tissue; (2) IPEH is often closely associated with a thrombus; (3) IPEH lacks necrosis; and (4) IPEH has limited mitotic activity [5, 14, 16]. All these characteristics correspond to our case. Accurate histopathological diagnosis is crucial to prevent overtreatment due to suspicion of angiosarcoma. Among the eight reported cases of IPEH of the maxillary sinus, three were suspected to be malignant [9, 13, 15]. In two reports, malignancy was ruled out by perioperative biopsy [11, 13]. Through intraoperative frozen section analysis, we could also rule out malignancy and avoid overly aggressive treatment.

Several surgical approaches have been proposed for IPEH. Endoscopic excision has been reported in a few studies [7, 11, 14, 15], Table 1. The open approach using the Caldwell-Luc procedure has been described to access the maxillary sinus to eradicate the disease [5]. In the case of large lesions, a combined approach, open and endoscopic [13] or additional skull base repair by the neurosurgery team, may be performed to eradicate the disease safely [14]. We confirmed with preoperative CT and MRI that there was no bone destruction or infiltration in the lateral maxillary sinus or skull base. Furthermore, intraoperative frozen section analysis ruled out malignancy. We were able to entirely remove the tumor using endoscopic surgery alone, even though it was a large lesion.

**Table 1:** Review of all reported cases of IPEH of the maxillary sinus in the English literature

Author	Year of publication	Age/sex	Localization	Symptoms	Imaging	Preoperative embolization	Surgical technique
Stern et al. [5]	1991	17/M	R. maxillary sinus, ethmoid, nasal cavity	Frontal headaches, R. cheek pain, exophthalmos	CT	Not mentioned	Caldwell-Luc excision
Lancaster et al. [7]	1998	67/F	L. maxillary sinus, ethmoid	L. nasal obstruction, rhinorrhoea, postnasal discharge, cheek aching	CT	Not mentioned	Endoscopic excision
Wang et al. [11]	2009	42/M	L. maxillary sinus, ethmoid, frontal sinus, nasal cavity	L. nasal obstruction, rhinorrhoea, epistaxis, frontal headaches	CT, MRI	None	Endoscopic excision
D'Aguanno et al. [13]	2019	67/F	R. maxillary sinus, nasal cavity	R. cheek aching, rhinorrhoea, postnasal discharge	CT, MRI	R. internal maxillary artery	Caldwell-Luc, Endoscopic excision
Cooke et al. [14]	2020	28/M	R. maxillary sinus, Bil nasal cavity, Bil ethmoid sinus	Bil. Nasal obstruction, epistaxis, headaches, R. cheek pain, itchy eye	CT	Bil. Sphenopalatine artery	Endoscopic excision, Skull base repair
Voruz F et al. [15]	2020	46/M	L. maxillary sinus	L. nasal obstruction, bloody-serous rhinorrhoea	CT, MRI	Not mentioned	Endoscopic excision
Voruz F et al. [15]	2020	76/M	R. maxillary sinus, nasal cavity	Epistaxis, rhinorrhoea	CT, MRI	Not mentioned	Endoscopic excision
Voruz F et al. [15]	2020	33/F	R. maxillary sinus	Rhinorrhoea, orbital pressure, headaches	CT, MRI	Not mentioned	Endoscopic excision
Present case	2023	58/M	L. maxillary sinus, Bil nasal cavity, Bil ethmoid sinus	Bil. Nasal obstruction, epiphora, epistaxis	CT, MRI	None	Endoscopic excision

*IPEH* Intravascular papillary endothelial hyperplasia, *M* male, *F* female, *R* right, *L* left, *Bil* bilateral, *CT* Computed tomography, *MRI* magnetic resonance imaging

The aetiology of IPEH has not yet been fully elucidated; however, many investigators have suggested that changes in the thrombotic process can lead to lesion development [3]. Any potential underlying cofactors promoting IPEH formation should be explored, given the diverse presentations in different cases. Recurrence of IPEH following incomplete excision of the lesion was documented as a result of poor exposure and visualization of the lesion has been reported [4, 6]. For postoperative follow-up of sinonasal IPEH, transnasal endoscopy is useful. Additionally, there is a possibility of submucosal recurrence, so if symptoms of bleeding or pain appear, an MRI should be performed. This report adds to the limited literature on IPEH by demonstrating its potential to mimic malignant processes and the critical role of accurate diagnosis through multidisciplinary collaboration. Complete resection remains a definitive treatment option. Recognizing this rare condition may prevent unnecessary overtreatment.

## Data Availability

No data was used for the research described in the article.
